# Indices of peripheral leukocytes predict longer overall survival in breast cancer patients on eribulin in Japan

**DOI:** 10.1007/s12282-021-01232-1

**Published:** 2021-03-07

**Authors:** Masato Takahashi, Kenichi Inoue, Hirofumi Mukai, Takashi Yamanaka, Chiyomi Egawa, Yasuo Miyoshi, Yukinori Sakata, Kenzo Muramoto, Hiroki Ikezawa, Toshiyuki Matsuoka, Junji Tsurutani

**Affiliations:** 1 Department of Breast Surgery, National Hospital Organization Hokkaido Cancer Center, 2-3-54, Kikusuishijo, Shiroishi-ku, Sapporo, Hokkaido 003-0804 Japan; 2 Division of Breast Oncology, Saitama Cancer Center, Kitaadachi-gun, Saitama Japan; 3 Division of Breast and Medical Oncology, National Cancer Center Hospital East, Kashiwa, Chiba Japan; 4 Department of Breast and Endocrine Surgery, Kanagawa Cancer Center, Yokohama, Kanagawa Japan; 5 Department of Breast Surgery, Kansai Rosai Hospital, Amagasaki, Hyogo Japan; 6 Division of Breast and Endocrine Surgery, Department of Surgery, Hyogo College of Medicine, Nishinomiya, Hyogo Japan; 7 Eisai Co., Ltd., Bunkyo-ku, Tokyo Japan; 8 Advanced Cancer Translational Research Institute, Showa University, Shinagawa-ku, Tokyo Japan; 9 Department of Medical Oncology, Kindai University, Osaka-Sayama, Osaka Japan

**Keywords:** Breast neoplasms, Eribulin, Japan, Product surveillance, post-marketing, Survival

## Abstract

**Background:**

It was reported that eribulin regulates the tumor microenvironment, including the immune system, by inducing vascular remodeling. Lymphocyte counts are a critical index of immune response in patients. The non-Asian, global EMBRACE study has suggested that baseline absolute lymphocyte count (ALC) may be a predictor of the survival benefit of eribulin in breast cancer patients. We examined whether the baseline ALC is a potential predictor of overall survival (OS) in Japanese patients with HER2-negative advanced breast cancer treated with eribulin.

**Methods:**

This was a post hoc analysis of data from a post-marketing observational study of eribulin in Japan. The OS by baseline ALC was estimated using the Kaplan–Meier method, with the cut-off value of 1500/μL for ALC. The OS by baseline neutrophil-to-lymphocyte ratio (NLR), a general prognostic index in breast cancer patients, was also estimated, with the cut-off value of 3.

**Results:**

The median OS was longer in patients with an ALC of ≥ 1500/μL than in those with an ALC of < 1500/μL (19.4 vs. 14.3 months; hazard ratio [HR]: 0.628; 95% confidence interval [CI]: 0.492, 0.801). Patients with an NLR of ≥ 3 showed shorter OS than those with an NLR of < 3 (13.2 vs. 18.8 months; HR: 1.552; 95% CI 1.254, 1.921), and NLR also separated OS in patients with an ALC of < 1500/μL.

**Conclusions:**

Consistent with the findings of a previous study involving a non-Asian, Western population, our study suggested that baseline ALC may be a predictive factor for the survival benefit of eribulin in Japanese patients.

**Supplementary Information:**

The online version contains supplementary material available at 10.1007/s12282-021-01232-1.

## Introduction

Eribulin mesylate (Halaven^®^, Eisai Co. Ltd., Tokyo, Japan) is a non-taxane microtubule dynamics inhibitor that was approved for the treatment of inoperable or recurrent breast cancer in Japan in 2011, regardless of previous treatment with other chemotherapy regimens. In the global phase 3 EMBRACE study, the overall survival (OS) was significantly prolonged in patients treated with eribulin as compared to that in patients receiving treatment of physician’s choice (TPC) [1]. The efficacy and safety of eribulin were also demonstrated in previous pre-approval and real-world studies [2, 3]. To assess the safety and effectiveness of eribulin in clinical settings in Japan, a post-marketing observational study was conducted in patients with HER2-negative advanced breast cancer, which showed favorable results consistent with the results of clinical trials and real-world studies [4, 5]. Despite the accumulated evidence on eribulin, the mechanisms of eribulin for efficacy outcomes are not fully understood; however, recent studies have suggested that the status of the tumor microenvironment may be the key to elucidate those [6, 7].

It has been hypothesized that the prolonged survival effect of eribulin may be attributable to its immunomodulatory effects; by reversing epithelial-to-mesenchymal transition (EMT), eribulin induces vascular remodeling [8–12] and improves the tumor microenvironment [13]. Generally, hypoxia suppresses immune regulation by inducing the expression of programmed death-ligand 1 (PD-L1) and transforming growth factor-beta (TGF-β) via the transcription factor hypoxia-inducible factor (HIF)-1. HIF-1 is also involved in the regulation of EMT. We speculated that eribulin regulates immune microenvironments and EMT by inducing vascular remodeling in breast cancer. A local immune reaction against cancer cells may be reflected in blood-based parameters, such as absolute lymphocyte count (ALC) and neutrophil-to-lymphocyte ratio (NLR). Indeed, these are reported as immune-related prognostic factors in patients with malignancy [14, 15]. High ALC and low NLR are reported as predictors of survival in patients with breast cancer treated with eribulin [6, 7, 16, 17]. However, the EMBRACE study suggested that ALC is an independent predictor of OS in eribulin-treated patients, whereas NLR is a general prognostic factor for improved OS [7].

To confirm whether the results of the EMBRACE study, which did not include Asian patients, hold true regardless of ethnicity, we performed a post hoc analysis and examined whether the baseline ALC and NLR are potential predictors of OS in Japanese patients treated with eribulin using data from a real-world post-marketing observational study in Japan [5].

## Materials and methods

### Study design

This was a post hoc analysis of data from a previous post-marketing study of eribulin in Japan to confirm whether the baseline ALC is a predictive marker for survival in eribulin-treated patients with breast cancer in Japan. This study aimed to confirm whether the results of the non-Asian, global EMBRACE study [7] hold true regardless of ethnicity. The details of the original post-marketing study of eribulin have been reported previously [4, 5]. In this multicenter, prospective, post-marketing, observational study in Japan (ClinicalTrials.gov: NCT02371174), patients were enrolled from September 2014 to February 2016 and were followed for a maximum of 2 years.

The scientific and ethical validity of the study design was reviewed by Eisai Co., Ltd. This study was conducted in accordance with the Declaration of Helsinki and Japanese Good Post-Marketing Study Practice (GPSP), an authorized standard for post-marketing surveillance. Under GPSP regulations, approvals from the institutional review boards of each institution or informed consent from participating patients are not mandatory; in practice, some institutions might have obtained approval or informed consent when deemed necessary. The personal data related to this study were managed in compliance with privacy protection laws in Japan.

### Patients

Eribulin-naïve patients with HER2-negative inoperable or recurrent breast cancer who received eribulin as first-/second-line or as third-/later-line chemotherapy were recruited in each institution in approximately equal numbers (1:1 ratio). Patients with severe bone marrow suppression (defined as a neutrophil count of < 1,000/mm^3^ or platelet count of < 75,000/mm^3^), patients with a history of hypersensitivity to eribulin components, and pregnant or possibly pregnant patients were excluded.

In accordance with the package insert, eribulin was administered intravenously at a standard dose of 1.4 mg/m^2^ for 2–5 min on day 1 (initiation of eribulin treatment; baseline) and day 8 of a 21-day cycle as indicated. The starting dose of eribulin was reduced (1.1 mg/m^2^) depending on the patient’s condition to prevent toxicity.

In this post hoc analysis, data on patients with available data on the baseline ALC and NLR (details are defined below) were analyzed.

### ALC and NLR data

The baseline ALC and NLR were measured using blood samples collected during 7 days before eribulin administration. Data closest to the date of eribulin administration, but before baseline were used in the analysis. NLR was calculated by dividing the absolute neutrophil count by ALC.

### Definitions

OS was defined as the time from the first eribulin administration to all-cause death or the last date on which the patient was known to be alive (censored).

The cut-off value of ALC was set at 1500/μL. A baseline ALC of < 1500/μL was defined as low, and a baseline ALC of ≥ 1500/μL was defined as high, in line with the previous study [7]. The cut-off value of NLR was set at 3. A baseline NLR of < 3 was defined as low, and a baseline NLR of ≥ 3 was defined as high, in line with previous studies [6, 7].

### Statistical analyses

All analyses were performed with data from patients with evaluable baseline ALC and NLR in the effectiveness analysis set in the post-marketing study. The data of patients without baseline blood test data were excluded. The baseline characteristics and eribulin treatment status were summarized descriptively using a baseline ALC cut-off value of 1500/μL and a baseline NLR cut-off value of 3.

First, to analyze the OS according to the baseline ALC or NLR, Kaplan–Meier curves were drawn to depict the survival rates in each group (i.e., ALC ≥ 1500 vs. < 1500/μL and NLR ≥ 3 vs. < 3) and the median OS (95% confidence interval [CI]) was estimated. The survival rates for 6 months, 1, and 2 years were also calculated. The hazard ratios (HRs) and 95% CIs for ALC of ≥ 1500 vs. < 1500/μL, and NLR of ≥ 3 vs. < 3 were estimated using the Cox proportional hazard model.

Although the ALC and NLR may individually be useful immune response markers, the combination of these markers may better predict survival outcomes in patients than each marker alone. Thus, to further examine the OS by combining the baseline ALC and NLR, patients were categorized into four groups: (1) ALC of ≥ 1500/μL and NLR of < 3, (2) ALC of ≥ 1500/μL and NLR of ≥ 3, (3) ALC of < 1500/μL and NLR of < 3, and (4) ALC of < 1500/μL and NLR of ≥ 3. The group of patients with an ALC of ≥ 1500/μL and an NLR of < 3 was used as a reference, and the HRs of the other groups were estimated.

To explore the relationship between ALC and NLR at baseline and the number of chemotherapy regimens for inoperable or metastatic disease prior to eribulin administration, HRs were estimated for ALC of ≥ 1500 vs. < 1500/μL and NLR of ≥ 3 vs. < 3 in each subgroup categorized by the number of chemotherapy regimens.

To explore the potential factors affecting OS, including the baseline ALC and NLR, univariate and multivariate Cox regression analyses were performed. First, HR and 95% CI were calculated for each factor using the univariate model. To further evaluate the factors affecting OS after adjusting for covariates, multivariate Cox regression analysis was performed using the stepwise selection method with a selection criterion of *p* < 0.20. Factors with *p* < 0.05 were considered statistically significant. Because the analysis was conducted for exploratory purposes, no adjustments were made for multiple testing. To best replicate the analysis of the potential factors for OS in the EMBRACE study, we planned to include the same factors with the same categories as in the previous study [7], as long as they were available in our data. Consequently, the following factors were considered in the univariate and multivariate Cox regression analyses: visceral metastasis, age, Eastern Cooperative Oncology Group Performance Status (ECOG PS), number of previous chemotherapy regimens for inoperable or metastatic disease, triple-negative, previous capecitabine treatment, estrogen receptor (ER) status, progesterone receptor (PgR) status, hormone receptor (HR) status, number of involved organs, baseline ALC, and baseline NLR.

Additionally, we also analyzed the OS of eribulin-treated patients using different ALC cut-off values by calculating the median OS and HR with 95% CIs for the high and low ALC groups using cut-off values ranging from 1000 to 1800/µL. This analysis was conducted to confirm whether a consistent trend (i.e., patients with a higher baseline ALC showed a longer OS than those with a lower ALC) was observed when using a different cut-off value instead of determining the optimal cut-off value for Japanese patients. All analyses were performed using SAS software version 9.4 (SAS Institute, Inc., Cary, NC, USA).

## Results

### Patient characteristics

**Fig. 1:**
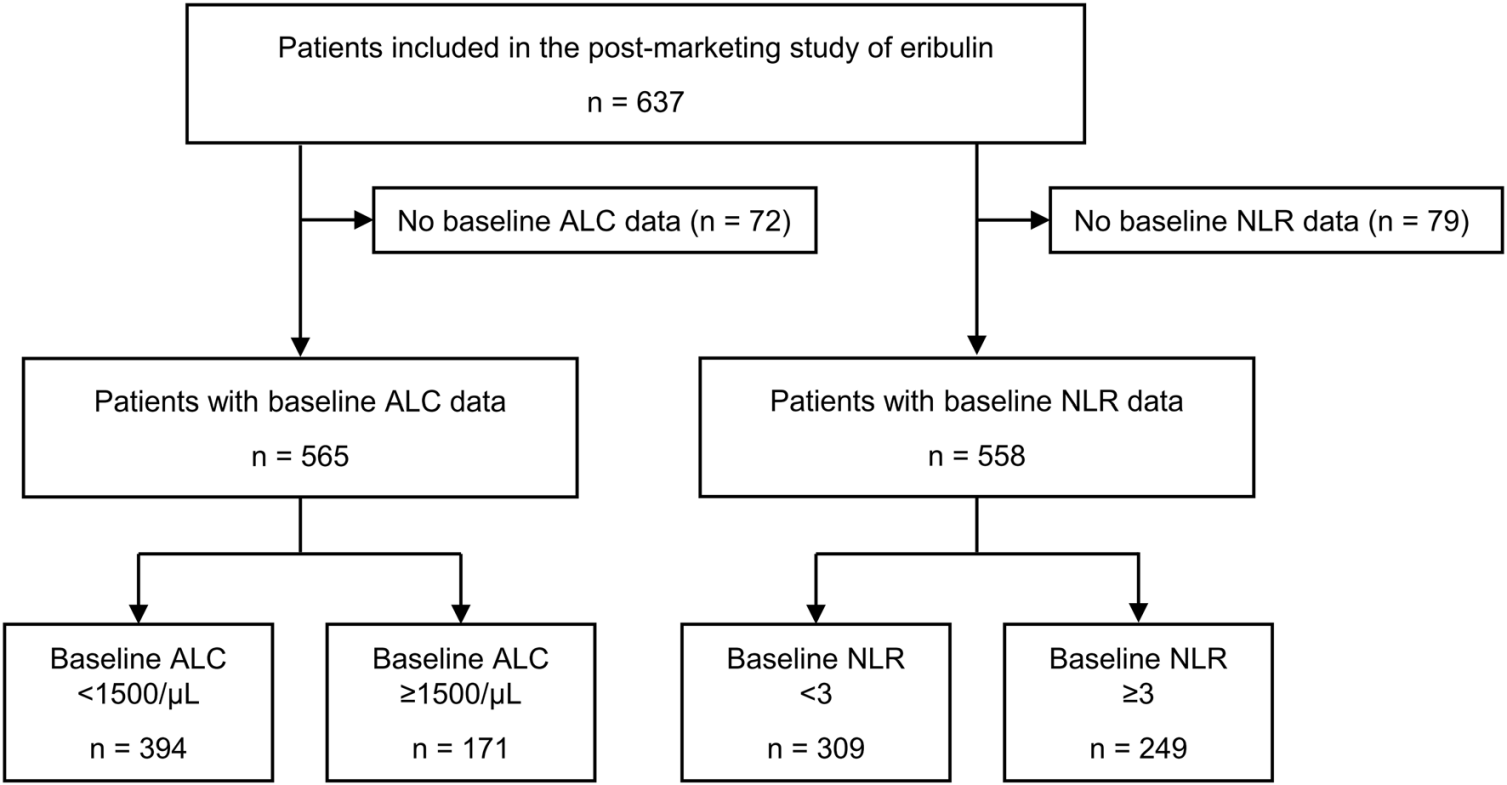
Flow diagram of patient selection. *ALC* absolute lymphocyte count, *NLR* neutrophil-to-lymphocyte ratio

Of the 637 patients analyzed in the original post-marketing observational study of eribulin, 565 patients with available baseline ALC data and 558 patients with available NLR data were included in the present post hoc analysis (Fig. 1). At baseline, 394 patients had an ALC of < 1500/μL and 171 patients had an ALC of ≥ 1500/μL; 309 patients had an NLR of < 3 and 249 patients had an NLR of ≥ 3 (Table 1). The patient characteristics were similar between those with an ALC of < 1500 and ≥ 1500/μL, and between those with an NLR of < 3 and ≥ 3. For instance, there were 248 (62.9%) patients aged < 65 years with a baseline ALC of < 1500/μL and 93 (54.4%) with a baseline ALC of ≥ 1500/μL; and 182 (58.9%) patients aged < 65 years with a baseline NLR of < 3 and 155 (62.2%) with a baseline NLR of ≥ 3. The proportion of patients with an ECOG PS of 0 was 54.8% in those with an ALC of < 1500/μL, 56.7% in those with an ALC of ≥ 1500/μL, 59.5% in those with an NLR of < 3, and 50.6% in those with an NLR of ≥ 3. The duration from recurrence to the administration of eribulin and the proportion of patients who underwent pre-operative or post-operative chemotherapy were also similar between the subgroups according to the baseline ALC or NLR.

**Table 1:** Patient characteristics by baseline ALC and NLR

Characteristics, *n* (%)^a^	ALC < 1500/μL (*n* = 394)	ALC ≥ 1500/μL (*n* = 171)	NLR < 3 (*n* = 309)	NLR ≥ 3 (*n* = 249)
Age				
< 65 years	248 (62.9)	93 (54.4)	182 (58.9)	155 (62.2)
≥ 65 years	146 (37.1)	78 (45.6)	127 (41.1)	94 (37.8)
ECOG PS				
0	216 (54.8)	97 (56.7)	184 (59.5)	126 (50.6)
1	149 (37.8)	65 (38.0)	112 (36.2)	98 (39.4)
2	25 (6.3)	8 (4.7)	12 (3.9)	21 (8.4)
3	4 (1.0)	1 (0.6)	1 (0.3)	4 (1.6)
4	0 (0.0)	0 (0.0)	0 (0.0)	0 (0.0)
HR assay				
No	7 (1.8)	3 (1.8)	4 (1.3)	6 (2.4)
Yes	387 (98.2)	168 (98.2)	305 (98.7)	243 (97.6)
ER status				
Positive	272 (69.0)	132 (77.2)	224 (72.5)	173 (69.5)
Negative	113 (28.7)	36 (21.1)	80 (25.9)	69 (27.7)
PgR status				
Positive	207 (52.5)	96 (56.1)	162 (52.4)	136 (54.6)
Negative	178 (45.2)	71 (41.5)	141 (45.6)	106 (42.6)
HR status				
Positive	279 (70.8)	132 (77.2)	226 (73.1)	178 (71.5)
Negative	106 (26.9)	36 (21.1)	78 (25.2)	64 (25.7)
Unknown	9 (2.3)	3 (1.8)	5 (1.6)	7 (2.8)
Triple-negative (HER2-negative, ER-negative, PgR-negative)				
No	279 (70.8)	132 (77.2)	226 (73.1)	178 (71.5)
Yes	106 (26.9)	36 (21.1)	78 (25.2)	64 (25.7)
Unknown	9 (2.3)	3 (1.8)	5 (1.6)	7 (2.8)
Metastases				
No	2 (0.5)	4 (2.3)	2 (0.6)	3 (1.2)
Yes	392 (99.5)	167 (97.7)	307 (99.4)	246 (98.8)
Affected side breast	26 (6.6)	9 (5.3)	17 (5.5)	18 (7.2)
Healthy side breast	12 (3.0)	5 (2.9)	7 (2.3)	10 (4.0)
Regional lymph node	112 (28.4)	50 (29.2)	95 (30.7)	65 (26.1)
Distal lymph node	124 (31.5)	49 (28.7)	89 (28.8)	83 (33.3)
Lung	154 (39.1)	68 (39.8)	121 (39.2)	99 (39.8)
Liver	197 (50.0)	69 (40.4)	139 (45.0)	123 (49.4)
Bone	231 (58.6)	90 (52.6)	159 (51.5)	157 (63.1)
Brain	36 (9.1)	5 (2.9)	11 (3.6)	29 (11.6)
Skin	56 (14.2)	20 (11.7)	32 (10.4)	44 (17.7)
Others	91 (23.1)	36 (21.1)	59 (19.1)	65 (26.1)
Time from recurrence to eribulin administration, years				
*n*	319	132	242	204
Mean ± SD	2.5 ± 3.3	3.0 ± 4.0	2.6 ± 3.6	2.7 ± 3.5
Median [range]	1.0 [0‒18]	1.0 [0‒19]	1.0 [0‒19]	1.0 [0‒18]
Pre-operative chemotherapy^b^				
No	229 (68.0)	108 (76.1)	180 (70.6)	153 (70.2)
Yes	98 (29.1)	31 (21.8)	67 (26.3)	60 (27.5)
Unknown	10 (3.0)	3 (2.1)	8 (3.1)	5 (2.3)
Post-operative chemotherapy^b^				
No	164 (48.7)	72 (50.7)	125 (49.0)	107 (49.1)
Yes	168 (49.9)	68 (47.9)	126 (49.4)	108 (49.5)
Unknown	5 (1.5)	2 (1.4)	4 (1.6)	3 (1.4)
Number of prior chemotherapy regimens for inoperable or metastatic disease				
0	77 (19.5)	46 (26.9)	64 (20.7)	55 (22.1)
1	112 (28.4)	47 (27.5)	96 (31.1)	62 (24.9)
2	97 (24.6)	38 (22.2)	74 (23.9)	60 (24.1)
3	47 (11.9)	18 (10.5)	34 (11.0)	31 (12.4)
4	35 (8.9)	16 (9.4)	25 (8.1)	26 (10.4)
≥ 5	25 (6.3)	6 (3.5)	16 (5.2)	14 (5.6)
Unknown	1 (0.3)	0 (0.0)	0 (0.0)	1 (0.4)
* n*	393	171	309	248
Mean ± SD	1.8 ± 1.5	1.6 ± 1.4	1.7 ± 1.5	1.8 ± 1.5
Median [range]	2.0 [0‒7]	1.0 [0‒6]	1.0 [0‒7]	2.0 [0‒6]
Previous capecitabine treatment				
No	337 (85.5)	148 (86.5)	265 (85.8)	213 (85.5)
Yes	57 (14.5)	23 (13.5)	44 (14.2)	36 (14.5)
Number of organs involved				
0	2 (0.5)	4 (2.3)	2 (0.6)	3 (1.2)
1	77 (19.5)	41 (24.0)	77 (24.9)	41 (16.5)
2	125 (31.7)	56 (32.7)	101 (32.7)	78 (31.3)
≥ 3	190 (48.2)	70 (40.9)	129 (41.7)	127 (51.0)
*n*	394	171	309	249
Mean ± SD	2.6 ± 1.3	2.3 ± 1.2	2.4 ± 1.2	2.8 ± 1.4
Median [range]	2.0 [0‒7]	2.0 [0‒7]	2.0 [0‒7]	3.0 [0‒7]
Baseline NLR				
< 3	160 (40.6)	149 (87.1)	‒	‒
≥ 3	230 (58.4)	19 (11.1)	‒	‒
Baseline ALC				
< 1500/μL	‒	‒	160 (51.8)	230 (92.4)
≥ 1500/μL	‒	‒	149 (48.2)	19 (7.6)

*ALC* absolute lymphocyte count, *ECOG PS* Eastern Cooperative Oncology Group Performance Status, *ER* estrogen receptor, *HER2* human epidermal growth factor receptor-2, *HR* hormone receptor, *NLR* neutrophil-to-lymphocyte ratio, *PgR* progesterone receptor, *SD* standard deviation

^a^Otherwise stated

^b^Proportions were calculated using the number of patients who received operative therapy as a denominator, namely, *n* = 337 (ALC < 1500/μL), *n* = 142 (ALC ≥ 1500/μL), n = 255 (NLR < 3), and *n* = 218 (NLR ≥ 3)

The mean duration of eribulin treatment ± standard deviation (SD) was longer in patients with a baseline ALC of ≥ 1500/μL than in those with a baseline ALC of < 1500/μL (31.8 ± 25.8 weeks and 23.9 ± 21.6 weeks, respectively) and in patients with a baseline NLR of < 3 than in those with a baseline NLR of ≥ 3 (29.5 ± 24.4 weeks and 22.3 ± 20.7 weeks, respectively).

### Effect of baseline ALC (cut-off value, 1500/μL) and NLR (cut-off value, 3) on OS

**Fig. 2:**
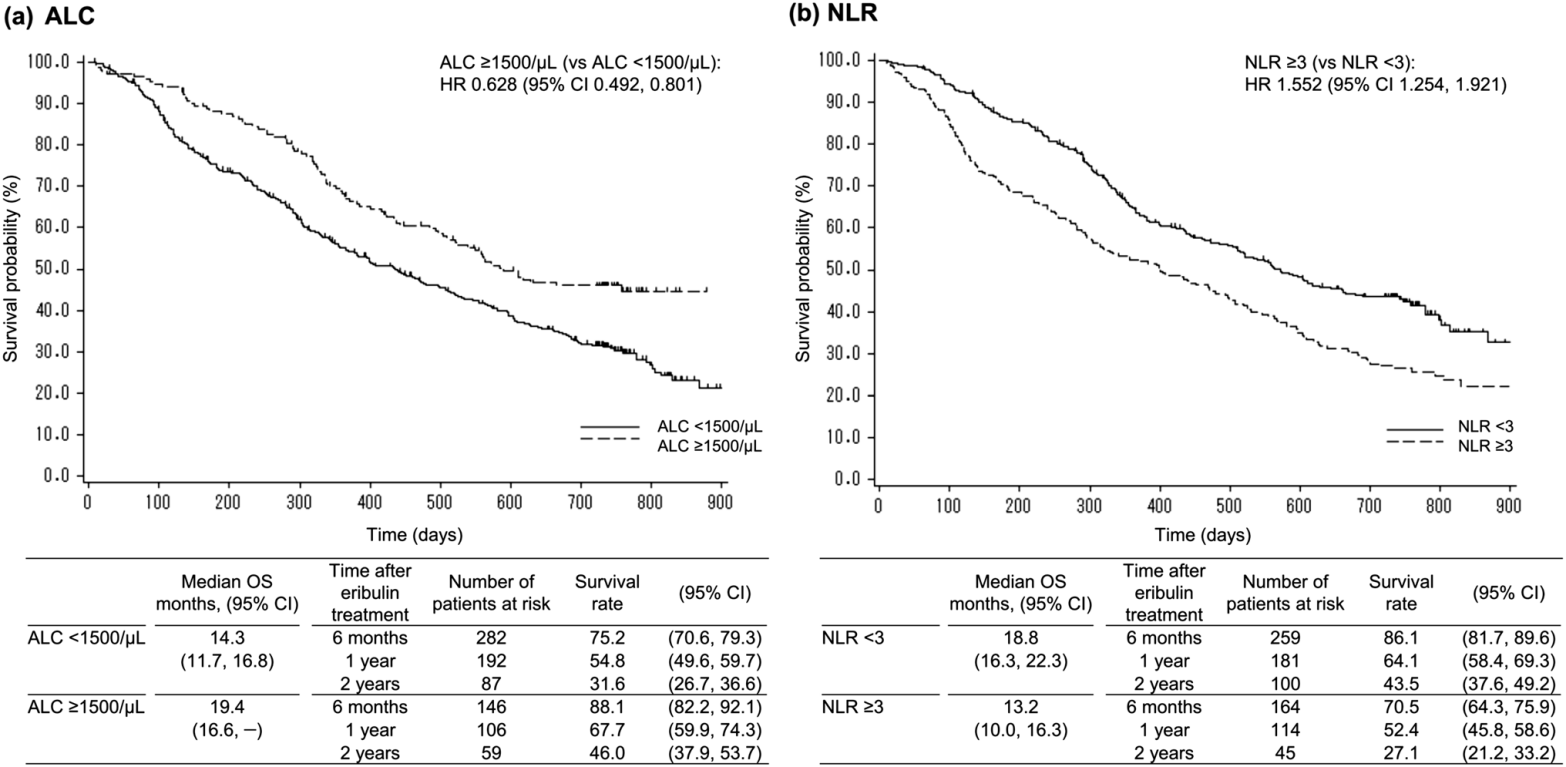
Overall survival by baseline ALC (< 1500 vs. ≥ 1500/μL) and NLR (< 3 vs. ≥ 3). *ALC* absolute lymphocyte count, *CI* confidence interval, *HR* hazard ratio, *NLR* neutrophil-to-lymphocyte ratio, *OS* overall survival

Figure 2 shows the results of OS based on the baseline ALC and NLR. The median OS (95% CI) was longer in patients with an ALC of ≥ 1500/μL as compared to that in patients with an ALC of < 1500/μL (19.4 [16.6, –] months vs. 14.3 [11.7, 16.8] months; HR: 0.628 [0.492, 0.801]) (Fig. 2a). For baseline NLR, the median OS (95% CI) was shorter in patients with an NLR of ≥ 3 than in those with an NLR of < 3 (13.2 [10.0, 16.3] months vs. 18.8 [16.3, 22.3] months; HR: 1.552 [1.254, 1.921]) (Fig. 2b).

### Combination analysis of baseline ALC and NLR values

**Fig. 3:**
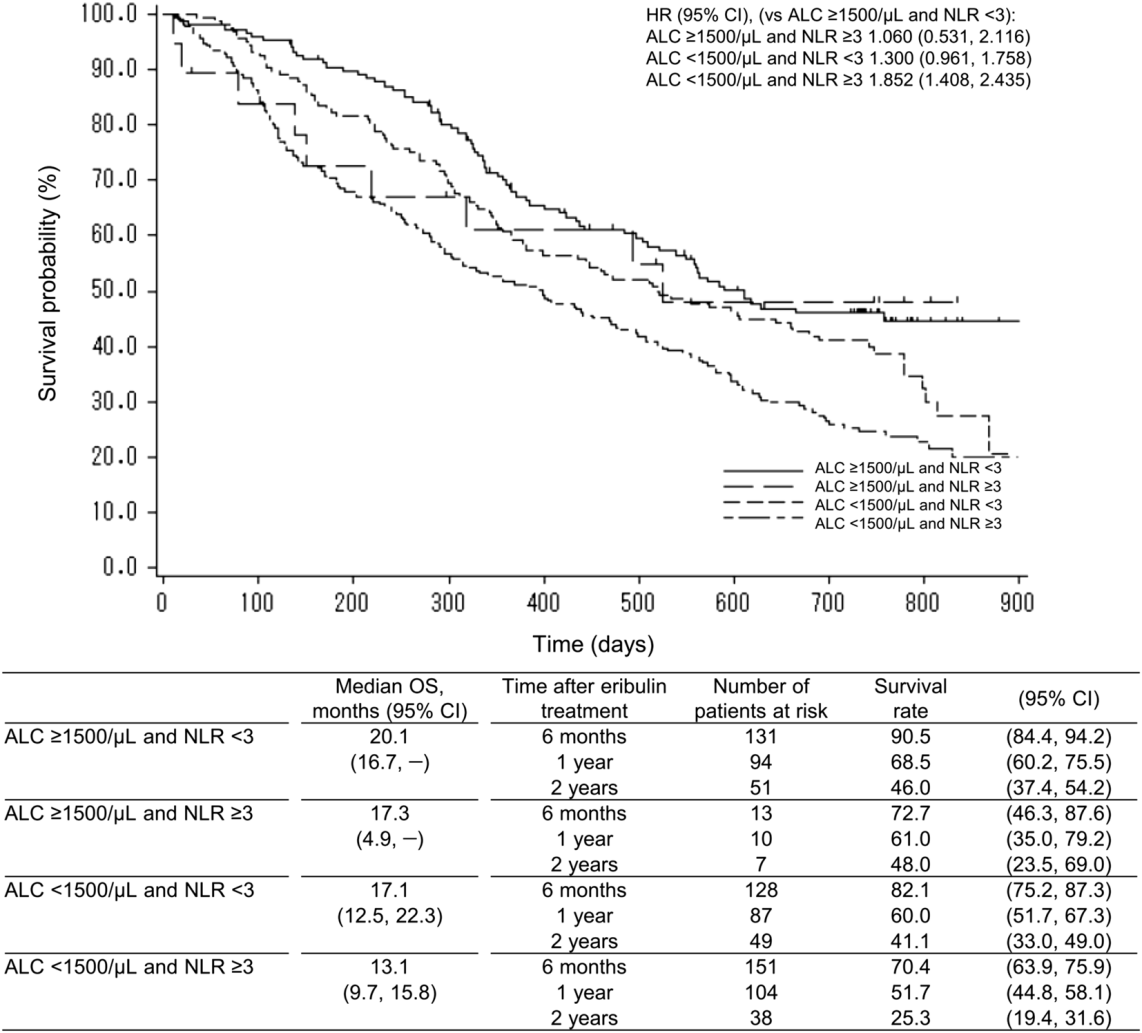
Overall survival by baseline ALC and NLR groups. *ALC* absolute lymphocyte count, *CI* confidence interval, *HR* hazard ratio, *NLR* neutrophil-to-lymphocyte ratio, *OS* overall survival

The patients were divided into four groups based on a combination of baseline ALC and NLR: (1) ALC of ≥ 1500/μL and NLR of < 3, (2) ALC of ≥ 1500/μL and NLR of ≥ 3, (3) ALC of < 1500/μL and NLR of < 3, and (4) ALC of < 1500/μL and NLR of ≥ 3 (Fig. 3). The median OS (95% CI) was highest in patients with an ALC of ≥ 1500/μL and an NLR of < 3 (20.1 months [16.7, –]) and lowest in patients with an ALC of < 1500/μL and an NLR of ≥ 3 (13.1 months [9.7, 15.8]). Using patients with an ALC of ≥ 1500/μL and an NLR of < 3 as a reference, the HR (95% CI) was 1.060 (0.531, 2.116) in patients with an ALC of ≥ 1500/μL and an NLR of ≥ 3, 1.300 (0.961, 1.758) in patients with an ALC of < 1500/μL and an NLR of < 3, and 1.852 (1.408, 2.435) in patients with an ALC of < 1500/μL and an NLR of ≥ 3.

### Effect of the number of chemotherapy regimens for inoperable or metastatic disease on OS

Irrespective of the number of chemotherapy regimens, the median OS (95% CI) was longer in patients with a baseline ALC of ≥ 1500/μL and an NLR of < 3 as compared to that in patients with an ALC of < 1500/μL and an NLR of ≥ 3 (ALC of ≥ 1500 vs. < 1500/μL, 31.0 vs. 19.9 months, 20.3 vs. 15.3 months, and 17.1 vs. 12.0 months; NLR of < 3 vs. ≥ 3, 26.4 vs. 19.9 months, 19.9 vs. 14.4 months, and 16.6 vs. 10.8 months in patients with eribulin as first-line treatment, second-line treatment, and third- or later-line treatment, respectively) (Supplementary Figure S1).

### Univariate and multivariate Cox regression analyses: factors affecting OS

Table 2 shows the results of the univariate and multivariate Cox regression analyses for factors affecting OS. The univariate Cox regression analysis identified ECOG PS, number of prior chemotherapy regimens for inoperable or metastatic disease, ER status, number of involved organs, visceral metastasis, baseline ALC, and baseline NLR (*p* < 0.001 for all). In the multivariate Cox regression analysis, the following six factors were retained in the model after a stepwise selection, all of which were found to be significant independent factors affecting OS: ECOG PS, PgR status, number of involved organs, visceral metastasis, baseline ALC, and baseline NLR.

**Table 2:** Univariate and multivariate Cox regression: factors affecting overall survival

Factor	Category		Univariate Cox regression				Multivariate Cox regression^b^			
			*n*	HR	95% CI	*p* value	*n*	HR	95% CI	*p* value
Age	< 65 years		378	1.121	(0.916, 1.373)	0.268	329			
	≥ 65 years	Ref	254				211			
ECOG PS	0		357	0.501	(0.411, 0.611)	< 0.001	300	0.490	(0.392, 0.612)	< 0.001
	≥ 1	Ref	275				240			
Number of previous chemotherapy regimens for inoperable or metastatic disease^a^	> 3	Ref	91				80			
	≤ 3		540	0.593	(0.458, 0.768)	< 0.001	460			
Triple-negative	No	Ref	458				398			
	Yes		157	1.423	(1.139, 1.777)	0.002	142			
Previous capecitabine treatment	No	Ref	541				461			
	Yes		91	1.156	(0.880, 1.519)	0.298	79			
ER status	Negative	Ref	164				149			
	Positive		451	0.663	(0.533, 0.825)	< 0.001	391			
PgR status	Negative	Ref	270				244			
	Positive		343	0.753	(0.616, 0.920)	0.006	296	0.611	(0.489, 0.763)	< 0.001
HR status	Negative	Ref	157				142			
	Positive		458	0.703	(0.563, 0.878)	0.002	398			
Number of organs involved	≥ 3	Ref	285				245			
	< 3		347	0.555	(0.455, 0.677)	< 0.001	295	0.744	(0.595, 0.932)	0.010
Visceral metastasis	No	Ref	61				51			
	Yes		571	2.625	(1.691, 4.075)	< 0.001	489	2.304	(1.405, 3.777)	< 0.001
Baseline ALC	< 1500/μL	Ref	390				377			
	≥ 1500/μL		170	0.628	(0.492, 0.801)	< 0.001	163	0.739	(0.562, 0.972)	0.031
Baseline NLR	< 3		306	0.644	(0.520, 0.797)	< 0.001	301	0.777	(0.611, 0.989)	0.040
	≥ 3	Ref	247				239			

*ALC* absolute lymphocyte count, *CI* confidence interval, *ECOG PS* Eastern Cooperative Oncology Group Performance Status, *ER* estrogen receptor, *HR* hazard ratio, *HR* hormone receptor, *NLR* neutrophil-to-lymphocyte ratio, *PgR* progesterone receptor, *SD* standard deviation

^a^Pre- and post-operative adjuvant chemotherapy regimens were not included

^b^All factors in this table were considered in the multivariate Cox regression analysis. After a stepwise selection, the ECOG PS, PgR status, number of organs involved, visceral metastasis, baseline ALC, and baseline NLR were retained in the model

### The cut-off value of ALC as a predictive effect of eribulin on OS

With different cut-off values of baseline ALC (1000–1800/μL), patients with a baseline ALC of ≥ 1500/μL showed the longest OS (19.4 months) and lowest HR (0.628) as compared to those with other cut-off values (Supplementary Table S1). Regardless of the cut-off values, patients with a higher baseline ALC showed longer OS (17.3‒19.4 months) than in those with a lower baseline ALC (14.2‒15.5 months).

## Discussion

In this post hoc analysis of post-marketing study data, we analyzed the OS according to the baseline ALC or NLR in eribulin-treated patients with breast cancer in Japan using the same cut-off values as those described in the previous EMBRACE study, in which Japanese patients were not included and most patients received later-line treatment [7]. In the present analysis, patients with a baseline ALC of ≥ 1500/μL and an NLR of < 3 showed prolonged OS as compared to those with an ALC of < 1500/μL and an NLR of ≥ 3. These results were consistent with those of the EMBRACE study (OS in this study vs. the EMBRACE study: 19.4 vs. 15.6 months in patients with an ALC of ≥ 1500/μL; 14.3 vs. 11.6 months in those with an ALC of < 1500/μL; 18.8 vs. 15.9 months in those with an NLR of < 3; and 13.2 vs. 10.5 months in those with an NLR of ≥ 3) [7]. Since this study included patients on early line treatment, particularly those on first-line treatment, the OS in this study was longer than that in the EMBRACE study. Furthermore, the multivariate Cox regression analysis revealed that both baseline ALC of ≥ 1500/μL and NLR of < 3 were independent factors for prolonged OS. The present study demonstrated that baseline ALC and NLR are prognostic markers for Japanese patients with HER2-negative advanced breast cancer treated with eribulin, including those on first-line treatment (Supplementary Figure S1).

Previous studies have reported inconsistent results regarding NLR as an independent predictor of survival in patients treated with eribulin. Ueno et al. reported that ALC and NLR were independent predictors of progression-free survival (PFS) [18]. Some studies have reported that NLR was associated with clinical response such as PFS [6, 19]. However, some other studies have suggested that NLR may not be an independent predictor of survival (e.g., OS and PFS) [7, 16]. One single-center retrospective study in Spain reported that the prognostic impact of NLR may be confounded by other factors, such as early stage of breast cancer, Asian race, and good ECOG PS (0–1) [20]. Similarly, the EMBRACE study, which included non-Asian patients with locally recurrent or metastatic breast cancer with a comparator group (TPC), reported that, irrespective of the treatment group, patients with a low NLR showed favorable OS results than those with a high NLR. This further suggested that ALC is a survival predictor in eribulin-treated patients but NLR is a general prognostic factor [7]. In the present study, when patients with an ALC of < 1500/μL were categorized as either an NLR < 3 or an NLR ≥ 3, the median OS was 17.1 and 13.3 months, respectively. Because NLR is a general prognostic index in breast cancer patients, we speculated that NLR affected the OS of patients with an ALC of < 1500/μL.

Regarding the cut-off value of ALC, a value of ≥ 1500/μL might be sufficient in predicting the survival outcome, given that the multivariate analysis identified an ALC of ≥ 1500/μL as a significant prognostic factor for OS in this study and the previous analysis [7]. Our study demonstrated that a cut-off value of 1500/μL had the lowest HR. However, patients with a higher ALC showed significantly longer OS than those with a lower ALC regardless of cut-off values from 1000 to 1800/uL, and the differences in results among different cut-off values were not large. Our results indicate that ALC is an appropriate and useful prognostic index; however, it is difficult to set an optimal cut-off value for ALC, because it might be affected by individual patient characteristics.

Our results also demonstrated that eribulin-treated patients with a high ALC before treatment showed longer OS. The results of studies on immune checkpoint inhibitors (ICIs) also demonstrated that ALC was associated with the treatment outcomes of ICIs [21, 22]. Ku et al. reported a trend of improved OS in the high- vs. low-baseline ALC group in a study on melanoma patients treated with ipilimumab (median OS, 13.3 vs. 5.1 months; *p* = 0.06) [23]. They concluded that the baseline ALC was an immunological predictive index for OS when treated with ICIs. Considering the aspect of ALC for predicting the efficacy of ICIs, ALC may reflect a potential immune response in patients. The authors hypothesized that lymphopenia may reflect a state of T-cell dysfunction resulting from immune exhaustion and depletion of anti-tumor lymphocytes and that these dysfunctions of lymphocytes have a limited ability to exert an anti-tumor effect in the setting of ICI therapy. In the case of eribulin, it was reported that PD-1, PD-L1, and FOXP3 levels were decreased, while the infiltrated CD8 + T-cell count was increased in a group that responded to eribulin; however, these findings were not observed in the non-responder group [13]. Furthermore, eribulin induced reoxygenation by vascular remodeling in advanced breast cancer patients, and TGF-β level, which is typically associated with hypoxic conditions, was decreased in the eribulin-treated group [10]. Recently, Kashiwagi et al. reported that the response to eribulin treatment was significantly associated with ALC. Furthermore, the expression of phosphorylated SMAD, induced by TGF-β, was significantly decreased by eribulin, especially in eribulin treatment responders [24]. It was found that hypoxia induced an increased expression of PD-L1, via the HIF-1 transcription factor, on the myeloid-derived suppressor cells [18]. HIF-1 also regulated TGF-β having potent immunosuppressive effects [25]. Taken together, we speculate that the longer OS in eribulin-treated patients is associated with modulation of the tumor microenvironment, including the immune regulation system, and the baseline ALC is a potential immunological marker and a beneficial biomarker in patients with HER2-negative advanced breast cancer treated with eribulin, because eribulin exerts immunomodulatory effects via vascular remodeling.

The interpretation of the results of this study may require careful consideration. First, because the target population was patients with HER2-negative advanced breast cancer in Japan, the results may not be generalizable to other populations, such as HER2-positive patients of a different race. Second, there might be unrecognized factors affecting OS with eribulin, including the confounders that were not evaluated in the post hoc analysis, even though we included the factors that were analyzed in the EMBRACE study [7]. In addition, the results of multiple Cox regression analysis should be considered exploratory, since no adjustments were made for multiple testing. Because we are at the hypothesis-generating stage, previous studies on ALC and NLR, as prognostic predictors for patients treated with eribulin, are limited. Under such circumstances, we explored the effect of ALC and NLR in a relatively large number of patients with advanced breast cancer in Japan, analyzing > 500 patients with evaluable ALC and NLR at baseline. This study is of great importance to further understand the effect of ALC and NLR on survival outcomes with eribulin in a real-world setting. Third, this analysis was performed to evaluate the effects of baseline ALC and NLR, thus, without considering these parameters post eribulin treatment. Furthermore, although the dose of eribulin did not largely differ between patients with a baseline ALC of < 1500/μL and those with a baseline ALC of ≥ 1500/μL, a greater proportion of patients with a baseline ALC of ≥ 1500/μL received an initial eribulin dose of 1.4 mg/m^2^ (standard dose); they were also treated with a slightly higher mean relative dose intensity (data not shown). Hence, it is possible that patients with a higher ALC at baseline received a higher dose of eribulin, which might have contributed to the improved OS in these patients compared to that in patients with a lower ALC.

In conclusion, in this post hoc analysis of a real-world post-marketing observational study in Japan, patients with HER2-negative advanced breast cancer receiving eribulin with a baseline ALC of ≥ 1500/μL and an NLR of < 3 showed prolonged OS as compared to those with an ALC of < 1500/μL and an NLR of ≥ 3. However, considering the previous results, the baseline ALC might serve as a predictive index for eribulin, and the baseline NLR is a general prognostic index in breast cancer patients. Thus, in line with the results of the previous EMBRACE study, an ALC of ≥ 1500/μL at baseline may be a predictor of survival in Japanese breast cancer patients receiving eribulin. Because ALC is a simple and easily assessable marker, it may be clinically useful in the optimal selection of patients with HER2-negative advanced breast cancer who may achieve a beneficial OS result from eribulin treatment.

## Supplementary Information

Below is the link to the electronic supplementary material.

Supplementary file1 (PDF 374 KB)

## References

[1] Cortes J, O'Shaughnessy J, Loesch D, Blum JL, Vahdat LT, Petrakova K (2011). Eribulin monotherapy versus treatment of physician's choice in patients with metastatic breast cancer (EMBRACE): a phase 3 open-label randomised study. Lancet.

[2] Kaufman PA, Awada A, Twelves C, Yelle L, Perez EA, Velikova G (2015). Phase III open-label randomized study of eribulin mesylate versus capecitabine in patients with locally advanced or metastatic breast cancer previously treated with an anthracycline and a taxane. J Clin Oncol.

[3] Watanabe J, Ito Y, Ohsumi S, Mizutani M, Tashiro H, Sakurai K (2017). Safety and effectiveness of eribulin in Japanese patients with locally advanced or metastatic breast cancer: a post-marketing observational study. Invest New Drugs.

[4] Tsurutani J, Sakata Y, Matsuoka T (2019). Chemotherapy-induced peripheral neuropathy in breast cancer patients treated with eribulin: interim data from a post-marketing observational study. Breast Cancer.

[5] Inoue K, Takahashi M, Mukai H, Yamanaka T, Egawa C, Sakata Y (2020). Effectiveness and safety of eribulin in Japanese patients with HER2-negative, advanced breast cancer: a 2-year post-marketing observational study in a real-world setting. Invest New Drugs.

[6] Miyagawa Y, Araki K, Bun A, Ozawa H, Fujimoto Y, Higuchi T (2018). Significant association between low baseline neutrophil-to-lymphocyte ratio and improved progression-free survival of patients with locally advanced or metastatic breast cancer treated with eribulin but not with Nab-paclitaxel. Clin Breast Cancer.

[7] Miyoshi Y, Yoshimura Y, Saito K, Muramoto K, Sugawara M, Alexis K (2020). High absolute lymphocyte counts are associated with longer overall survival in patients with metastatic breast cancer treated with eribulin-but not with treatment of physician's choice-in the EMBRACE study. Breast Cancer.

[8] Yoshida T, Ozawa Y, Kimura T, Sato Y, Kuznetsov G, Xu S (2014). Eribulin mesilate suppresses experimental metastasis of breast cancer cells by reversing phenotype from epithelial-mesenchymal transition (EMT) to mesenchymal–epithelial transition (MET) states. Br J Cancer.

[9] Funahashi Y, Okamoto K, Adachi Y, Semba T, Uesugi M, Ozawa Y (2014). Eribulin mesylate reduces tumor microenvironment abnormality by vascular remodeling in preclinical human breast cancer models. Cancer Sci.

[10] Ueda S, Saeki T, Takeuchi H, Shigekawa T, Yamane T, Kuji I (2016). In vivo imaging of eribulin-induced reoxygenation in advanced breast cancer patients: a comparison to bevacizumab. Br J Cancer.

[11] Ito K, Hamamichi S, Abe T, Akagi T, Shirota H, Kawano S (2017). Antitumor effects of eribulin depend on modulation of the tumor microenvironment by vascular remodeling in mouse models. Cancer Sci.

[12] Zhao S, Yu W, Ukon N, Tan C, Nishijima KI, Shimizu Y (2019). Elimination of tumor hypoxia by eribulin demonstrated by 18F-FMISO hypoxia imaging in human tumor xenograft models. EJNMMI Res.

[13] Goto W, Kashiwagi S, Asano Y, Takada K, Morisaki T, Fujita H (2018). Eribulin promotes antitumor immune responses in patients with locally advanced or metastatic breast cancer. Anticancer Res.

[14] Ray-Coquard I, Cropet C, Van Glabbeke M, Sebban C, Le Cesne A, Judson I (2009). Lymphopenia as a prognostic factor for overall survival in advanced carcinomas, sarcomas, and lymphomas. Cancer Res.

[15] Koh CH, Bhoo-Pathy N, Ng KL, Jabir RS, Tan GH, See MH (2015). Utility of pre-treatment neutrophil-lymphocyte ratio and platelet-lymphocyte ratio as prognostic factors in breast cancer. Br J Cancer.

[16] Araki K, Ito Y, Fukada I, Kobayashi K, Miyagawa Y, Imamura M (2018). Predictive impact of absolute lymphocyte counts for progression-free survival in human epidermal growth factor receptor 2-positive advanced breast cancer treated with pertuzumab and trastuzumab plus eribulin or nab-paclitaxel. BMC Cancer.

[17] Watanabe J, Saito M, Horimoto Y, Nakamoto S (2020). A maintained absolute lymphocyte count predicts the overall survival benefit from eribulin therapy, including eribulin re-administration, in HER2-negative advanced breast cancer patients: a single-institutional experience. Breast Cancer Res Treat.

[18] Ueno A, Maeda R, Kin T, Ito M, Kawasaki K, Ohtani S (2019). Utility of the absolute lymphocyte count and neutrophil/lymphocyte ratio for predicting survival in patients with metastatic breast cancer on eribulin: a real-world observational study. Chemotherapy.

[19] Myojin M, Horimoto Y, Ito M, Kitano S, Ishizuka Y, Sasaki R (2020). Neutrophil-to-lymphocyte ratio and histological type might predict clinical responses to eriburin-based treatment in patients with metastatic breast cancer. Breast Cancer.

[20] Ivars Rubio A, Yufera JC, de la Morena P, Fernández Sánchez A, Navarro Manzano E, García Garre E (2019). Neutrophil-lymphocyte ratio in metastatic breast cancer is not an independent predictor of survival, but depends on other variables. Sci Rep.

[21] Diehl A, Yarchoan M, Hopkins A, Jaffee E, Grossman SA (2017). Relationships between lymphocyte counts and treatment-related toxicities and clinical responses in patients with solid tumors treated with PD-1 checkpoint inhibitors. Oncotarget.

[22] Muto Y, Kitano S, Tsutsumida A, Namikawa K, Takahashi A, Nakamura Y (2019). Investigation of clinical factors associated with longer overall survival in advanced melanoma patients treated with sequential ipilimumab. J Dermatol.

[23] Ku GY, Yuan J, Page DB, Schroeder SE, Panageas KS, Carvajal RD (2010). Single-institution experience with ipilimumab in advanced melanoma patients in the compassionate use setting: lymphocyte count after 2 doses correlates with survival. Version 2. Cancer.

[24] Kashiwagi S, Asano Y, Goto W, Takada K, Morisaki T, Kouhashi R (2020). Validation of systemic and local tumour immune response to eribulin chemotherapy in the treatment of breast cancer. Anticancer Res.

[25] Taylor MA, Lee YH, Schiemann WP (2011). Role of TGF-beta and the tumor microenvironment during mammary tumorigenesis. Gene Expr.

