# Supplementary material for: Indices of peripheral leukocytes predict longer overall survival in breast cancer patients on eribulin in Japan
Source: Breast Cancer. 2021 Mar 7;28(4):945–55. doi: 10.1007/s12282-021-01232-1 (PMC8213560; doi:10.1007/s12282-021-01232-1)
Supplement: Supplementary file 1 — Supplementary file1 (PDF 374 KB) [file 12282_2021_1232_MOESM1_ESM.pdf]

Supplementary File

Article title: Indices of peripheral leukocytes predict longer overall survival in breast cancer patients on eribulin in Japan

Journal name: *Breast Cancer*

Author names: Masato Takahashi, Kenichi Inoue, Hirofumi Mukai, Takashi Yamanaka, Chiyomi Egawa, Yasuo Miyoshi, Yukinori Sakata, Kenzo Muramoto, Hiroki Ikezawa, Toshiyuki Matsuoka, Junji Tsurutani

Corresponding author:
Masato Takahashi
Department of Breast Surgery
National Hospital Organization Hokkaido Cancer Center
2-3-54, Kikusuishijo, Shiroishi-ku, Sapporo, Hokkaido 003-0804, Japan
Tel: +81-11-811-9111
Fax: +81-11-811-9153
E-mail: masato.takahashi0725@gmail.com

Table S1. Cutoff value of ALC as a predictor for the effect of eribulin on overall survival

ALC cutoff (/μL)	n	Median OS (months)	HR	95% CI (Lower)	95% CI (Upper)	*p* value
≥1000	359	17.3	0.695	0.560	0.862	*p<0.001*
<1000 (Ref.)	201	14.2				
≥1100	322	17.6	0.685	0.554	0.846	*p<0.001*
<1100 (Ref.)	238	14.2				
≥1200	274	18.5	0.641	0.517	0.793	*p<0.001*
<1200 (Ref.)	286	14.2				
≥1300	237	18.5	0.673	0.541	0.837	*p<0.001*
<1300 (Ref.)	323	14.3				
≥1400	196	18.8	0.695	0.552	0.873	*p =0.002*
<1400 (Ref.)	364	14.5				
≥1500	170	19.4	0.628	0.492	0.801	*p<0.001*
<1500 (Ref.)	390	14.3				
≥1600	144	18.5	0.691	0.537	0.891	*p =0.004*
<1600 (Ref.)	416	14.7				
≥1700	113	18.4	0.755	0.574	0.994	*p =0.044*
<1700 (Ref.)	447	15.5				
≥1800	89	18.8	0.726	0.537	0.983	*p =0.037*
<1800 (Ref.)	471	15.5				

ALC, absolute lymphocyte count; CI, confidence interval; HR, hazard ratio; NLR, neutrophil-to-lymphocyte ratio; OS, overall survival; Ref, reference.

1) Eribulin as first-line

(a) ALC

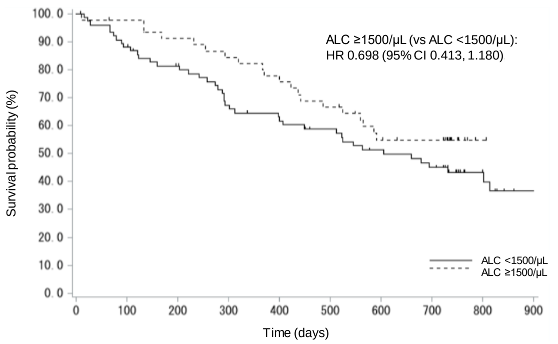

	Median OS months, (95% CI)	Time after eribulin treatment	Number of patients at risk	Survival rate	(95% CI)
ALC <1500/µL	19.9	6 months	60	81.4	(70.6, 88.5)
	(13.1, 26.8)	1 year	45	64.6	(52.4, 74.3)
		2 years	26	45.1	(33.1, 56.3)
ALC ≥1500/µL	31.0	6 months	41	91.2	(78.1, 96.6)
	(17.3, −)	1 year	37	82.3	(67.6, 90.7)
		2 years	18	54.9	(39.1, 68.1)

(b) NLR

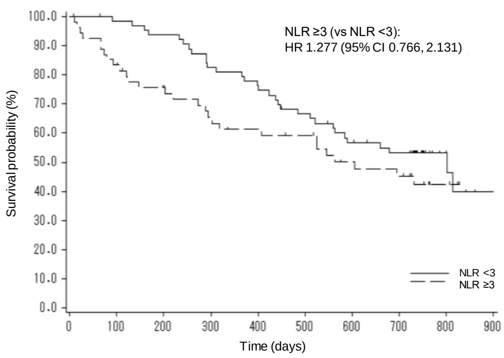

	Median OS months, (95% CI)	Time after eribulin treatment	Number of patients at risk	Survival rate	(95% CI)
NLR <3	26.4	6 months	59	93.7	(84.0, 97.6)
	(18.4, 31.0)	1 year	51	81.0	(68.9, 88.7)
		2 years	27	53.2	(40.0, 64.7)
NLR ≥3	19.9	6 months	39	75.6	(61.7, 85.1)
	(9.9, −)	1 year	29	61.3	(46.6, 73.1)
		2 years	16	45.3	(30.8, 58.7)

2) Eribulin as second-line

(a) ALC

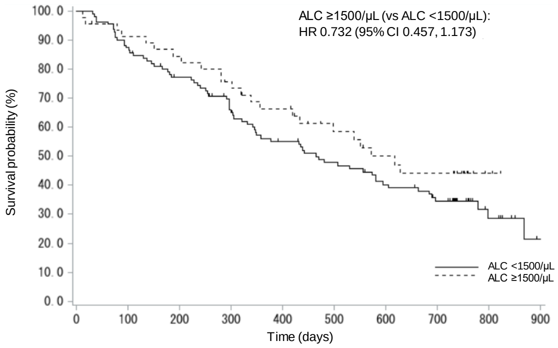

	Median OS months, (95% CI)	Time after eribulin treatment	Number of patients at risk	Survival rate	(95% CI)
ALC <1500/µL	15.3	6 months	86	79.2	(70.4, 85.7)
	(11.3, 19.6)	1 year	57	56.1	(46.2, 65.0)
		2 years	26	34.7	(25.4, 44.1)
ALC ≥1500/µL	20.3	6 months	39	86.8	(72.9, 93.8)
	(13.8, −)	1 year	28	66.3	(50.4, 78.2)
		2 years	15	44.1	(28.3, 58.9)

(b) NLR

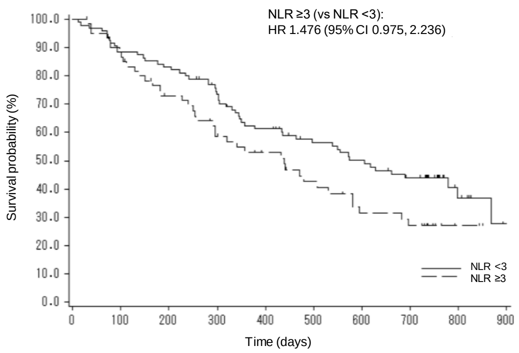

	Median OS months, (95% CI)	Time after eribulin treatment	Number of patients at risk	Survival rate	(95% CI)
NLR <3	19.9	6 months	80	84.2	(75.2, 90.2)
	(14.2, 26.2)	1 year	56	62.4	(51.7, 71.4)
		2 years	32	44.0	(33.3, 54.1)
NLR ≥3	14.4	6 months	44	76.5	(63.5, 85.3)
	(9.5, 19.1)	1 year	28	52.9	(39.2, 64.9)
		2 years	9	27.0	(15.5, 39.9)

3) Eribulin as third or later-line

(a) ALC

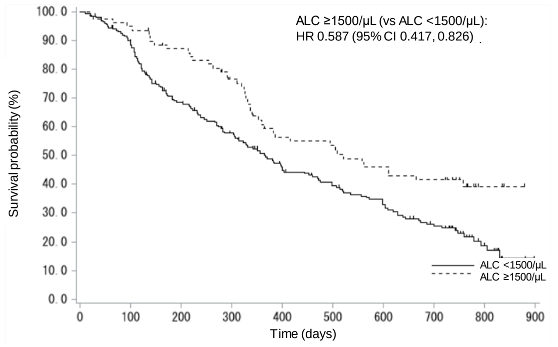

	Median OS months, (95% CI)	Time after eribulin treatment	Number of patients at risk	Survival rate	(95% CI)
ALC <1500/µL	12.0	6 months	135	70.5	(63.6, 76.4)
	(10.0, 15.1)	1 year	89	50.0	(42.7, 56.9)
		2 years	35	24.8	(18.6, 31.4)
ALC ≥1500/µL	17.1	6 months	66	87.1	(77.3, 92.8)
	(11.8, −)	1 year	41	59.4	(47.2, 69.6)
		2 years	26	41.6	(30.0, 52.8)

(b) NLR

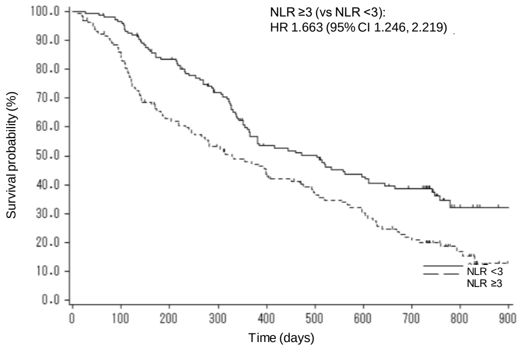

	Median OS months, (95% CI)	Time after eribulin treatment	Number of patients at risk	Survival rate	(95% CI)
NLR <3	16.6	6 months	120	84.1	(77.1, 89.2)
	(11.9, 20.1)	1 year	74	57.5	(48.7, 65.3)
		2 years	41	38.7	(30.3, 47.0)
NLR ≥3	10.8	6 months	80	65.4	(56.5, 73.0)
	(7.9, 14.9)	1 year	56	48.0	(39.0, 56.5)
		2 years	20	20.0	(13.2, 27.8)

Fig. S1 Overall survival by baseline ALC, NLR, and the number of regimens

ALC, absolute lymphocyte count; CI, confidence interval; HR, hazard ratio; NLR, neutrophil-to-lymphocyte ratio; OS, overall survival.
